# Cost-effectiveness of intermittent preventive treatment of malaria in infants (IPTi) for averting anaemia in Gabon: a comparison between intention to treat and according to protocol analyses

**DOI:** 10.1186/1475-2875-10-305

**Published:** 2011-10-17

**Authors:** Elisa Sicuri, Prosper Biao, Guy Hutton, Fabrizio Tediosi, Clara Menendez, Bertrand Lell, Peter Kremsner, Lesong Conteh, Martin P Grobusch

**Affiliations:** 1 Barcelona Centre for International Health Research, Hospital Clínic, Universitat de Barcelona, (Rosselló 132), Barcelona (08036), Spain; 2 Direction de la Programmation et de la Prosperctive (DPP), Ministère de la santé, Cotonou (05 BP 688), Bénin; 3 Development Solutions International GmbH, (95 Austrasse), Basel (4051), Switzerland; 4 Department of Institutional Analysis and Public Management, Centre for Research on Health and Social Care Management (CERGAS), Università Bocconi, (Via Roentgen 1), Milano (20136), Italy; 5 Centro de Investigação em Saúde de Manhiça (CISM), (Rua 12), Vila de Manhiça, Maputo (CP 1929), Mozambique; 6 Medical Research Unit, Albert Schweitzer Hospital, Lambaréné, Gabon; 7 Institute of Tropical Medicine, University of Tübingen, Tübingen (D-72074), Germany; 8 Institute of Global Health Innovation, Imperial College, London (SW7 2AZ), UK; 9 Center for Tropical Medicine and Travel Medicine, Academic Medical Center, University of Amsterdam, (Meibergdreef 9), Amsterdam (1100 DD), The Netherlands

## Abstract

**Background:**

In Gabon, the impact of intermittent preventive treatment of malaria in infants (IPTi) was not statistically significant on malaria reduction, but the impact on moderate anaemia was, with some differences between the intention to treat (ITT) and the according to protocol (ATP) trial analyses. Specifically, ATP was statistically significant, while ITT analysis was borderline. The main reason for the difference between ITT and ATP populations was migration.

**Methods:**

This study estimates the cost-effectiveness of IPTi on the reduction of anaemia in Gabon, comparing results of the ITT and the ATP clinical trial analyses. Threshold analysis was conducted to identify when the intervention costs and protective efficacy of IPTi for the ATP cohort equalled the ITT cost-effectiveness ratio.

**Results:**

Based on IPTi intervention costs, the cost per episode of moderate anaemia averted was US$12.88 (CI 95% 4.19, 30.48) using the ITT analysis and US$11.30 (CI 95% 4.56, 26.66) using the ATP analysis. In order for the ATP results to equal the cost-effectiveness of ITT, total ATP intervention costs should rise from 118.38 to 134 US$ ATP or the protective efficacy should fall from 27% to 18.1%. The uncertainty surrounding the cost-effectiveness ratio using ITT trial results was higher than using ATP results.

**Conclusions:**

Migration implies great challenges in the organization of health interventions that require repeat visits in Gabon. This was apparent in the study as the cost-effectiveness of IPTp-SP worsened when drop out from the prevention was taken into account. Despite such challenges, IPTi was both inexpensive and efficacious in averting cases of moderate anaemia in infants.

## Background

The relationship between malaria and anaemia is complex both in terms of how it can be explained in biomedical terms and as part of economic evaluations. Malaria and anaemia account for a major burden of disease among sub-Saharan children both independently and in combination [1]. Anaemia can be caused by many etiologic factors, including iron and other nutritional deficiencies. Malaria is clearly an important factor that may also lead to severe anaemia with a significant risk of death [2,3]. In areas of high malaria transmission, the risk of anaemia is highest in infants [4]. Prevention of malaria infection either by insecticide-treated nets (ITNs) or drugs (intermittent preventive treatment of malaria in infants - IPTi - or chemoprophylaxis) have been shown to significantly reduce infant anaemia in endemic countries [5-7].

Anaemia in children less than 5 years old is classified by the World Health Organization (WHO) into three categories, according to Haemoglobin (Hb) concentration in blood: severe (Hb ≤ 5 g/dl), moderate (5 <Hb < 8 g/dl) and mild (8 ≤ Hb < 11 g/dl). Mild anaemia can signal other problems associated with poor nutrition rather than being a health problem itself. No disability weights are associated with mild anaemia in the Global Burden of Disease [8]. Severe anaemia impairs growth, motor and mental development and increases the risk of child mortality [9]. Although the association between non-severe forms of anaemia and mortality is not entirely clear [10], mild to moderate anaemia has been found to be the cause of deficits in child development [11].

Anaemia is widespread among Gabonese children. A recent observational study showed that most children presenting to the health facility with severe falciparum malaria were less than five years of age (92.3% of 583 cases) and anaemia was the most frequent feature of severe malaria (67.8% of cases) [12]. In another observational study in Gabon 69.5% of the malaria-infected children aged from 0 to 10 years were moderately to severely anaemic, with infants at higher risk of developing severe malarial anaemia [13].

While a large number of clinical trials report the impact interventions targeting malaria can have on anaemia [14], very few economic evaluations report this effect [15,16], with only one attempting to model the cost-effectiveness of each malaria and anaemia [17]. Ideally a cost-effectiveness analysis would present the impact of an intervention on all the co-morbidities [18]. This, however, can be a challenge as disability adjusted life years (DALYs) do not readily lend themselves to understanding the effect of an intervention on multiple health outcomes [19]. While individual DALYs for different disease states can be added up, this may not be appropriate as co-morbidities are not necessarily the sum of separate DALY disease weightings [19,20]. Many economic evaluations, including the current one, rely on natural units such as episodes of a particular illness averted, but again, this does not provide insights into the aggregate cost-effectiveness of an intervention for more than one illness at a time.

In this article, anaemia was explored in the context of a randomized controlled trial of intermittent preventive treatment of malaria in infants (IPTi) in Gabon [21]. IPTi involves delivering preventive doses of an anti-malarial drug during routine Expanded Programme on Immunization (EPI) visits, regardless of plasmodial infection status [22,23]. More specifically, the cost-effectiveness of IPTi was investigated in terms of its impact on moderate anaemia among study infants with and without malaria. Pooled data from six trials (one each in Tanzania, Mozambique and Gabon, and three in Ghana) assessed the protective efficacy (PE) of IPTi with sulphadoxine-pyrimethamine (SP) to be 30.3% (CI 95% 19.8%, 39.4%, p < 0.0001) against clinical malaria, 21.3% (CI 95% 8.2%, 32.5%, p = 0.002) against the risk of anaemia, 38.1% (CI 95% 12.5%, 56.2%, p = 0.007) against hospital admissions associated with malaria parasitaemia, and 22.9% (CI 95% 10.0%, 34.0%, p = 0.001) against all-cause hospital admissions [14].

**Figure 1:**
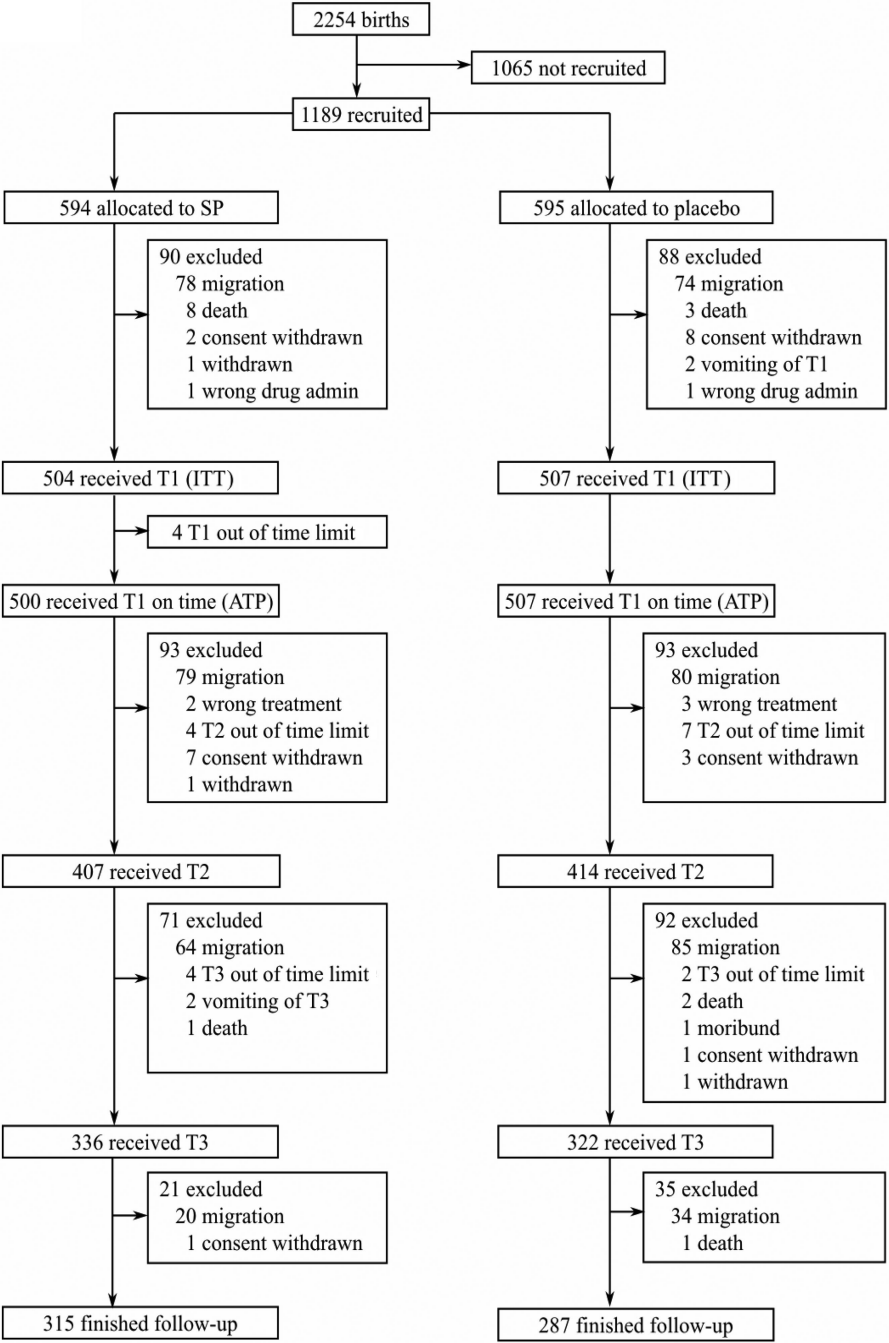
**Trial profile of intermittent preventive treatment of malaria in infants in Gabon**. **Notes**: Taken from Grobusch *et al *[21]; T1 = treatment 1, T2 = treatment 2, T3 = treatment 3

In the IPTi country specific trial, Gabonese infants received SP at 3, 9, 15 months (at the time of the third dose of diphtheria, pertussis and tetanus - DPT3 - and measles vaccines plus an extra visit at 15 months). The impact on malaria was not statistically significant, but the impact on anaemia was, with some differences between the intention to treat (ITT) and the according to protocol (ATP) analysis at 18 months of age [21]. Specifically, ATP was statistically significant, while ITT analysis was borderline, due in large part to migration of the infants enrolled in the trial (Figure 1). As IPTi was not statistically significant on malaria reduction (PE 17%; CI 95% -24%, 44%), the confidence interval of the incremental cost-effectiveness ratio (ICER) was extremely wide and with a wide negative portion, at US$29.94 (CI 95% -184.16, 208.75) per DALY averted based on intervention costs [24]. The statistically significant impact of IPTi on anaemia was not included, suggesting that the economic evaluation of IPTi in Gabon could have been underestimated. The cost-effectiveness analysis of IPTi in Gabon is presented by focusing on the impact of the prevention on the reduction of anaemia cases and comparing results based on the ITT and on APT analysis of the efficacy results of the trial at 18 months, that is, three months after the last dose intake.

## Methods

### Study population

Recruitment for the clinical trial this study refers to was undertaken at the maternity wards of the Albert Schweitzer Hospital (HAS) and of the General Hospital (HG) in Lambaréné, a town of about 25,000 inhabitants, situated in the Moyen-Ogooué province, in the western part of Gabon. Lambaréné is the main political, medical and economic centre of the province. The entomological inoculation rate in the area is 50 infective bites/person/year. Malaria transmission is perennial, with little seasonal variation. Bed net use, at least in the peripartal period, was 80% in the study population during IPTi trial. However, only a fraction of nets were impregnated, and insecticide-treated bed net (ITN) coverage was 5% [21].

### Efficacy and effectiveness

Mild, moderate and severe anaemia were defined in the trial according to WHO criteria. Children enrolled were given SP or placebo at 3, 9, and 15 months and both actively and passively followed up. Monthly home visits for health status assessment, until 30 months of age was reached, were carried out and in the case of an acute febrile disease, a finger-prick blood sample was obtained and a thick blood film examined. In addition, between home visits, parents were expected to present at the HAS research unit if the child experienced health problems. On day 0, before administration and on days 7 and 28 after each drug administration, clinical chemistry, full blood count, and thick blood smears were performed [21].

Trial data presented results according to the two different perspectives: the intention to treat (ITT) population and the according to protocol (ATP) population [21]. The modified ITT population was defined as all subjects who correctly received the first drug administration within the time limit of one month, regardless of the status of the other two scheduled administrations. Therefore, within the ITT population there were children who received one treatment, children who received two treatments and children who received the whole course of three treatments. The according to protocol (ATP) population only included subjects who received all three drug administrations within the specified time limits and were followed up until 18 months of age. As a result, 1,011 (85% of the recruited infants) received at least one dose correctly within the time limits (ITT population), and 602 (51% of the recruited infants) received three treatments correctly and were followed up for 18 months (ATP population). At 18 months the ITT analysis showed a PE of 22% (CI 95% -1%, 40%), p = 0.06 while the ATP analysis showed a PE of 27% (CI 95% 2%, 46%), p = 0.04 against at least one episode of moderate anaemia. This difference between ITT and ATP efficacies was certainly due to the distinct levels of adherence to the prevention. Although uneven drop-out between groups might have played a role, the lack of data did not allow to verify this hypothesis.

In order to avoid over-optimistic estimates of the efficacy of an intervention, there is currently a general consensus that economic evaluations of trials should be based on ITT analysis of data generated during clinical/epidemiological studies [25-29]. In most of the clinical trials, the primary assessment is based on ITT analysis and other types of analysis, such as ATP, are secondary and exploratory. Therefore, economic evaluations based on such secondary and exploratory analysis of trial data should be justified [25]. However, under particular circumstances related to the characteristics of the trial or in presence of discrepancies between how the intervention is being carried out during the trial and how it is likely to be implemented in practice, intention to treat analysis may bias the economic evaluation [26].

In the current study it was found relevant to explore how different definitions of efficacy may impact on the economic evaluation of the intervention. Such differences were substantial in the IPTi trial in Gabon, as ITT analysis led to non-statistically significant results and ATP analysis to statistically significant results of the intervention. In addition, such differences were not just a statistical artefact as they reflected a relevant socio-economic phenomenon in the area, such as migration. The authors were interested in assessing if and how such differences affected the ICERs. Specifically, to represent the different efficacies in the economic evaluation different ICERs were calculated for ITT and ATP results at 18 months.

In this study, efficacy is defined as percentage reduction in attack rates in the SP compared to the placebo group, e.g. protective efficacy of IPTi against moderate anaemia resulting from the trial. Effectiveness is defined as number of episodes of anaemia that could potentially be averted in real life conditions. Specifically, the number of episodes of moderate anaemia averted was calculated by multiplying the number of children receiving the prevention times the protective efficacy of IPTi against moderate anaemia times the malaria incidence in the placebo group.

### Costings

Intervention costs are presented from the perspective of the provider only, more specifically the cost to the health system of providing IPTi was estimated. Household costs associated with accessing IPTi were not included and thought to be negligible as infants were already assumed to engage with EPI services. Although there is research suggesting EPI is not always easily accessed [30].

The initial costings focused on treating malaria episodes, therefore, the potential savings to providers (the health system) and households achieved by averting anaemia inpatient or outpatient visits were not included. When comparing ICERs across different interventions, to limit bias, it is suggested that intervention costs alone are included, therefore not having included health system and household savings from fewer cases of anaemia should not be seen as a limitation [31]. The implications of this are discussed in more detail later.

### IPTi

The full economic costs of the intervention were based on a detailed costing of IPTi delivery in Mtwara, Southern Tanzania where over 13,976 infants were given IPTi over the course of two years as part of a phased implementation study delivering IPTi within routine health services across five districts [32]. A breakdown of the cost per dose of delivering SP in Mtwara is given elsewhere [33]. Costs included national and district costs associated with policy change, community sensitization, behaviour change and communication, drug purchase and distribution, training, administration of IPTi in health facilities and management. The costs of delivering IPTi in Mtwara were adapted to the Gabonese setting to allow for the costs of delivering IPTi in operational circumstances and not one generated in more artificial trial conditions of Gabon. For example, in Gabon the dispensing of IPTi was undertaken in the research centre separately from the main health facilities, therefore, adjustments had to be made to model the cost of IPTi within an EPI setting, based on Mtwara resource use. The SP drug prices from the Gabonese national government central medical stores were used.

Tradable and non-tradable components on the Southern Tanzanian IPTi study were inflated to US$ 2007 rates. Tradable components of the unit cost were inflated using US inflation rates. To work out the international dollar equivalent of buying the same amount of goods and services in Gabon as compared to Tanzania, the non-tradable components of the unit costs were adjusted based on international dollar differences using purchasing power parity (PPP) adjustment rates [34].

To allow for more accurate ITT and ATP unit costs, IPTi delivery was split into fixed and variable costs. Variable costs were associated with resources that were dependent on the number of IPTi doses received, and composed of drug purchase and distribution costs and administration costs of the intervention in the health facility. All other costs were considered to be fixed, that is, necessary costs independent of the number of doses actually administered to infants. Total cost of the intervention was calculated differently for the ITT and the ATP population. The trial enrolled 1,189 infants, of which 594 were randomly assigned to the SP group. The ITT population was composed of 1,011 infants (504 in the SP and 507 in the Placebo) while the ATP population, a sub-group of the ITT population, was composed of 602 infants (315 received SP and 287 Placebo), see Figure 1. Some infants dropped out from the study between the moment of recruitment and first dose intake. Infants were recruited and randomized into SP and control arms at birth and mothers were asked to take their children to the HAS research unit for visits at 3, 9 and 15 months. EPI vaccinations were given during the same visits. Of the 594 infants enrolled and randomized in the SP group, the 85% received at least one dose (N = 504), the 68.5% received at least two doses (N = 407) and the 53% received the whole course of three doses (N = 315). The 594 infants randomly assigned to the SP group were considered as if they were the target participants of IPTi, therefore, total costs for the target population of 594 infants were calculated according to the following formulae:

$$\begin{array}{c} \text{ITT population}=594*\text{unit fixed cost}+\left(504-407\right)*\text{unit variable cost}*1\text{ dose SP }\\ +\left(407-315\right)*\text{unit variable cost}*2\text{ doses SP}+315*\text{unit variable cost}*3\text{ doses SP} \end{array}$$

ATP population=594*unit fixed cost+315*unit variable cost*3 doses SP

All costs are presented in 2007 US$.

### Cost-effectiveness analysis

Having presented the cost-effectiveness of IPTi on malaria in Gabon elsewhere [24] below the cost-effectiveness of IPTi on anaemia is shown.

ICERs for IPTi were calculated for the ITT population (504 infants) and the ATP population (315 infants) by dividing costs of delivering IPTi by the number of episodes of anaemia averted. The number of episodes of anaemia averted in the ITT population was calculated by multiplying 504 times the protective efficacy calculated with reference to ITT population times the malaria incidence in the ITT placebo group. The number of episodes of anaemia averted in the ATP population was calculated by multiplying 315 times the protective efficacy calculated with reference to ATP population times the malaria incidence in the ATP placebo group.

All input variables of the cost-effectiveness model were expressed as probability distribution. Ranges for the triangular distributions assigned to each variable were taken from different sources (Table 1). Triangular distribution was used to be consistent with Hutton *et al *[31] and Conteh *et al *[24]. Where possible, input variables were analysed using their confidence intervals (such as protective efficacies). Where only point estimates were available it was necessary to make assumptions on their uncertainty. As in Sicuri *et al *[35], ranges were assumed to be equal to ±25% or ±50% of the estimated mean value. Wider ranges were assigned to allow for more variation to those factors presenting higher uncertainty in time and space within the country (e.g. incidence of malaria) [36] and narrower ranges were assigned to factors assumed to have a lower level of variation (e.g. intervention costs).

**Table 1:** Input variables used in the probabilistic cost-effectiveness analysis of IPTi-SP^a ^on anaemia

Variable	Details of the variables	Probability distribution	Source	Range
***IPTi-SP***^***a ***^ ***total provider costs in US$ 2007 for the ITT population***	Fixed costs to administer 3 doses IPTi-SP^a ^to a hypothetical target of 594 infants + variable costs to administer 1 dose IPTi-SP^a ^to an actual target of 97 infants, 2 doses to 92 infants and 3 doses to 315 infants	Triangular (97.70, 130.26, 162.82)	[24,33]	±25%

***IPTi-SP***^***a ***^ ***total provider costs US$ 2007 for the ATP population***	Fixed costs to administer 3 doses IPTi-SP^a ^to a hypothetical target of 594 infants + variable costs to administer 3 doses IPTi-SP^a ^to an actual target of 315 infants	Triangular (88.78, 118.38, 147.98)	[24,33]	±25%

**Cost-effectiveness analysis - input variables**				

***Intention to treat (ITT)***				

***Protective Efficacy 18 months***	Protective efficacy of IPTp-SP^a ^to avert anaemia at 18 months follow up (ITT analysis)	Triangular (-0.01,0.22,0.4)	[21]	[21]

***Malaria incidence***	Rate per person-years at risk in placebo group	Triangular (0.08,0.16,0.24)	[21]	±50%

***According to protocol (ATP)***				

***Protective Efficacy 18 months***	Protective efficacy of IPTp-SP^a ^to avert anaemia at 18 months follow up (ATP analysis)	Triangular (0.02, 0.27, 0.46)	[21]	[21]

***Malaria incidence***	Rate per person-years at risk in placebo group	Triangular (0.075, 0.15, 0.3)	[21]	±50%

^**a**^Intermittent preventive treatment of malaria in infants with sulphadoxine-pyrimethamine.

Probabilistic cost-effectiveness analysis was performed through Monte Carlo simulations using @Risk (version 5.0) add-in tool to Microsoft Excel^© ^(Palisade Corporation, Ithaca, NY, USA).

Threshold analysis was undertaken to compare under what circumstances ATP and ITT cost-effectiveness would produce the same cost-effectiveness ratio. Specifically, threshold analysis explored at what level of intervention costs and protective efficacy the ICER obtained through the ATP analysis equalled the ICER obtained through the more commonly used ITT analysis. Therefore, the ICER resulting from the mostly used ITT analysis was used as cut-off value for the threshold analysis of the ATP cost effectiveness, instead of using cut-off levels employed when ICERs are expressed in terms of DALYs averted [37].

## Results

### Costs

For a target population of 594 infants, total costs of the intervention were US$130.26 for the ITT population and US$118.38 for the ATP population, see Table 1. These costs were used as inputs in the cost-effectiveness model with a range of ±25% as minimum and maximum value of their assumed probability distribution.

### Cost-effectiveness analysis

As shown in Table 2, in the ITT analysis, the number of moderate anaemia episodes averted was 16.45 (CI 95% 4.35, 30.26) among 504 infants. The number of moderate anaemia episodes averted was 13.73 (CI 95% 4.70, 24.81) among 315 infants in the ATP analysis.

**Table 2:** Cost-effectiveness of IPTi-SP^a ^to prevent anaemia in Lambaréné, Gabon

Intervention costs per moderate anaemia cases averted			
	**US $ 2007**	**95% CI**^b^	**Min, Max**

Intention to treat	12.88	4.19, 30.48	-259.80, 1367.16

According to protocol	11.3	4.56, 26.66	2.97, 97.83

**Number of episodes of moderate anaemia averted**			

	**N**	**95% CI**^b^	**Min, Max**

Intention to treat	16.45	4.35, 30.26	-0.44, 42.22

According to protocol	13.73	4.70, 24.81	1.44, 36.73

^a ^Intermittent preventive treatment of malaria in infants with sulphadoxine-pyrimethamine [(Malaria episodes averted thanks to IPTi were 20 (CI 95% -20, 57) and intervention cost per malaria episode averted was US$ 11.93 (CI 95% -90.60, 102.87) [24]]

^b ^Confidence Interval

Using ITT efficacy, the cost per moderate anaemia episode averted was US$12.88 (CI 95% 4.19, 30.48). With reference to ATP efficacy, this was US$11.30 (CI 95% 4.56, 26.66). While both mean values and confidence intervals resulted were not very different in the two types of analysis, the distribution of the cost-effectiveness ratio relative to the ITT analysis were much more dispersed with a very low minimum and a very high maximum value of -259.80 and of 1367.16 US$ per episode averted, respectively (Table 2).

### Threshold analysis

**Figure 2:**
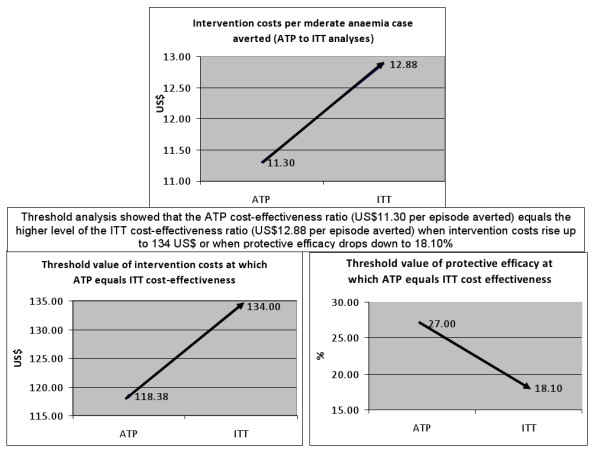
**Threshold analysis of the cost-effectiveness of IPTi comparing ATP and ITT results**. **Notes**: IPTi = intermittent preventive treatment of malaria in infants; ATP = According to Protocol; ITT = Intention to Treat

Threshold analysis showed that ATP cost-effectiveness ratio equals the higher level of the ITT cost-effectiveness ratio (US$12.88 per episode averted) when intervention costs rise up to US$134 or when protective efficacy drops down to 18.1%. In other words, the most cost-effective ATP analysis equals the least cost-effective results of the ITT analysis when ATP intervention costs increased from 118.38 to 134 US$ or when protective efficacy decreased from 27.00% to 18.1%. Figure 2 presents these results.

## Conclusions

The findings in this paper focus on two topics: (i) the cost-effectiveness of IPTi at averting anaemia and (ii) the influence of using ATP as the measure of effect as opposed to the more commonly used ITT.

Independently of the type of analysis used, this study assessed that the average cost of a first or only episode of moderate anaemia averted during infancy/early childhood is lower than US$13. Considering the high burden associated with anaemia in the country, this can be considered as a very valuable intervention. Specifically, the cost per case of anaemia averted in the ITT analysis was, on average, US$12.88. It is difficult to compare this finding to others which show an effect on anaemia as so few studies have looked at the cost-effectiveness of averting anaemia cases; and those which have, tend to use DALYs, not episodes, as their unit of effectiveness. The only other study the authors have identified using a similar approach to this study was an economic evaluation of IPT in schoolchildren undertaken in Kenya, in which the cost per case of anaemia averted was calculated at US$29.84 (2006 US$) [38].

Using both types of results, ITT and ATP, can give important information to policy makers. The main finding of this study is that although IPTp-SP is inexpensive and efficacious in reducing moderate anaemia, its cost-effectiveness worsens when drop-out is considered. This result may be intuitive but it is really important in a setting like Lambaréné where drop-out is primarily caused by migration and where any type of health intervention is likely to face the same challenge. The difference between ITT and ATP allows a comparison of the cost-effectiveness of IPTi-SP in two scenarios: one in which several of the initial recipients of the intervention move elsewhere and do not complete the programme (ITT); another, in which a smaller number of recipients complete the prevention programme (ATP). This difference has both cost and health system/organizational repercussion. The more cost-effective between the two was the ATP, meaning that total coverage and total adherence constitute the best situation. However, in a context of migration, reaching complete coverage and adherence is likely to imply high costs.

In this study, it was considered more appropriate to report ICERs in terms of the number of episodes of anaemia averted rather than in terms of DALYs averted. The magnitude of DALYs averted is particularly sensitive to long-term and severe disabilities and to the case fatality rate of a disease [39]. IPTi results in Gabon pointed to a reduction in non-severe cases of anaemia, which are likely to lead neither to long-term, severe disabilities nor to death. In addition, even if IPTi would have shown reductions of severe anaemia, which is associated to death, there is no evidence in Gabon that severe anaemia in children leads to death [29]. Without a sufficiently robust estimate of the anaemia case fatality rate, any DALY calculation is debatable.

IPTi was more cost-effective at preventing moderate anaemia when ATP results were used to measure both efficacy (protective efficacy of IPTi as outcome of the trial) and effectiveness (episodes of anaemia averted due to IPTi). ICERs in terms of intervention costs divided by protective efficacy of IPTi are not reported. However, when analysing *efficacy*, ATP intervention costs were lower than ITT intervention costs because the ATP population was a sub-group of the ITT population and, therefore, fewer infants were administered with the prevention. In addition, ATP efficacy was higher than ITT efficacy. Moving from efficacy to effectiveness, effectiveness was, on average, higher in the ITT than in the ATP population (ITT cases averted were 16.45; ATP cases averted were 13.73). This was due to the higher number of infants included in the ITT compared to the ATP population. ITT however, still remained less cost-effective than ATP due to the higher total intervention costs.

The threshold analysis was conducted to explore the extent that two influential ICER parameters could change in order for the cost-effectiveness of the ATP population to equal the more commonly used ITT population. Findings suggested that intervention costs should rise from 118.38 to 134.00 US$ for the ICER of the ATP analysis to equal the ITT one. Since both fixed unit costs and target population (594 infants) were assumed to be identical across both types of analysis, the variable that made the difference in determining total intervention costs was the number of infants actually administered with the three doses. For the ATP ICER to reach the ITT ICER, 435 infants instead of 315 would need to take all three doses in order for the intervention costs to rise up to 134.00 US$. However, the rise in the number of infants taking three doses would also have a positive effect on the protective efficacy of the intervention, thus affecting the cost-effectiveness ratio at the denominator.

The use of ATP efficacy reduced the uncertainty existing in the cost-effectiveness analysis, since ATP efficacy was statistically significant. This reduction in uncertainty was visible with reference to the minimum and the maximum value of the cost-effectiveness ratios (Table 2). Both in this analysis and in the one presented in Conteh *et al *[24], ICERs are based on borderline statistically significant and statistically non-significant primary outcomes from the clinical trial in Gabon [21]. There is some debate about the value of undertaking economic analysis based on non-significant clinical results. In accordance with Johnston *et al *[40], the authors believe it is worth performing economic evaluation with such clinical results. Cost minimization, where cost differences are reported without the calculation of an ICER, was traditionally used as response to non-significant efficacy differences of a trial. However, cost minimization is often inappropriate as it assumes the trial was powered to show equivalence of effects, which is rarely the case and it was not in IPTi trials [41]. Johnston *et al *[40] advocate that cost-effectiveness ratios should be calculated even in absence of significance of efficacy, but uncertainty surrounding ICERs needs to be quantified and highlighted.

While randomized controlled trials (RCTs) provide crucial information on the efficacy of an intervention (represented by the protective efficacy of IPTi in this study), they do not always provide clear information on the effectiveness (represented here by the actual number of moderate anaemia cases averted) as they are conducted under atypical conditions. However, RCTs can give some signs of what coverage might be in a real world situation. As to be expected, the health benefits that infants get from the three doses is higher than the benefits from fewer doses, however, it is evident that in Gabon adherence to three doses of IPTi spread out over 15 months will prove a challenge.

In Gabon, vaccination coverage, represented by DTP3 coverage, scheduled at 14 weeks was 14% in 1983, reached a maximum level of 70% in 1995 and dropped to 45% in 2009, ranking Gabon as the country with the fourth lowest DTP3 coverage in the African region (after Chad, Equatorial Guinea and Somalia) [42]. While migration became a relevant phenomenon from the early 1980s across Gabon and could have had an impact, the little information available on this topic does not explain the changes in coverage entirely [43]. In addition to migration, the fluctuating EPI coverage and the lack of the demand for vaccinations has been associated with side effects, attitudes towards vaccinations, limited access to providers and bad previous experiences at mother and child clinics. Gysels *et al *reported that in Gabon 80% (184/231) of respondents interviewed about their use of EPI services and perceptions of IPTi claimed that shame related to having sick or malnourished infants, having too many children, or being a young mother, were common reasons for avoiding routine clinic visits [44]. All these factors that inhibited demand for EPI may well have played a role in the fall in uptake of IPTi.

Although Conteh *et al *[24], Hutton *et al *[31] and this study are economic evaluations based on the same study trial, the aim of this study was different and subsequently the methodology followed differs. In Conteh *et al *and in Hutton *et al *the dropout from the intervention was calculated starting from the intake of the first dose of SP. In this study, in order to focus on the differences between ITT and ATP definitions of efficacy, the drop-out was calculated starting from one step prior to this. If IPTi was to be introduced, mothers would be sensitized about the intervention while in the health facility having just given birth, and would then be expected to start IPTi on a later occasion in line with their infant's vaccination schedule. To reflect this in our analysis, the drop-out rate was considered to start from when infants were initially recruited for the trial in the health facility having just given birth, three months before the administration of the first IPTi dose.

The IPTi Gabon trial was the only trial in which coverage dropped to such an extent between the enrolment and the first dose. Considering coverage loss from the target population to the first dose is important from a policy perspective. If preventive health interventions to infants, at local level, are planned to target newborns, such plans should take into account the potential migration of the target population. More research is needed to explore the migration patterns in Gabon and how these can shape an individual's treatment seeking behaviour.

Cost-effectiveness is an important tool when it comes to assessing an intervention for possible implementation. IPTi-SP has shown to be an additional malaria control tool of value [14] and has, consequently, recently been added to the WHO's malaria control instrument portfolio [45]. However, IPTi-SP is recognized to be of use in particular settings, i.e. in areas of high endemicity, stable transmission and low SP resistance. For a variety of reasons, such as the efficacy of IPTi bordering on significance [21,46] and high levels of SP resistance [47], IPTi-SP will most likely not be considered as an intervention of high priority in many countries. Before such country-level decisions are made, it should be noted that IPTi-SP can have positive effects in addition to averting malaria. Seasonal IPTi in areas of unstable transmission is under evaluation [48] and recent modelling has predicted IPTi can be cost effective even in settings of low transmission, seasonality, using short-acting and more expensive drugs and in conditions of increasing drug resistance [49]. In addition to averting malaria, the cost-effectiveness of IPTi at averting anaemia has been explored in this paper, and other evidence exists to suggest IPTi can have an impact on all cause admissions [14] and increase EPI vaccine uptake [50]. It is important therefore to assess any intervention, not only on the impact it has on the primary endpoint of a randomized controlled trial, but to also consider the often difficult to measure, wider, health benefits to the health system and the individual.

## Competing interests

The authors declare that they have no competing interests.

## Authors' contributions

ES and LC and MPG conceived of the study. The general cost-effectiveness model was originally conceived by GH and FT for the economic evaluation of IPTi trials. ES and LC analysed the data. ES, MPG and LC interpreted the data. ES, LC drafted the manuscript with substantial contribution from PB, GH, FT, CM, BL, PK and MPG. All authors have read and approved the final manuscript.
